# Metabolic Traits and Stroke Risk in Individuals of African Ancestry: Mendelian Randomization Analysis

**DOI:** 10.1161/STROKEAHA.121.034747

**Published:** 2021-06-03

**Authors:** Segun Fatumo, Ville Karhunen, Tinashe Chikowore, Toure Sounkou, Brenda Udosen, Chisom Ezenwa, Mariam Nakabuye, Opeyemi Soremekun, Iyas Daghlas, David K. Ryan, Amybel Taylor, Amy M. Mason, Scott M. Damrauer, Marijana Vujkovic, Keith L. Keene, Myriam Fornage, Marjo-Riitta Järvelin, Stephen Burgess, Dipender Gill

**Affiliations:** 1 The African Computational genomics (TACG) Research group, Medical Research Council/Uganda Virus Research Institute (MRC/UVRI) and London School of Hygiene and Tropical Medicine (LSHTM), Entebbe, Uganda (S.F., T.S., B.U., C.E., M.N., O.S.).; 2 Department of Non-communicable Disease Epidemiology (NCDE), London School of Hygiene and Tropical Medicine London, United Kingdom (S.F.).; 3 H3Africa Bioinformatics Network (H3ABioNet) Node, Centre for Genomics Research and Innovation, NABDA/FMST, Abuja, Nigeria (S.F., C.E.).; 4 MRC Biostatistics Unit, School of Clinical Medicine (S.F., S.B.), University of Cambridge, United Kingdom.; 5 Cardiovascular Epidemiology Unit, Department of Public Health and Primary Care (A.M.M., S.B.), University of Cambridge, United Kingdom.; 6 Department of Epidemiology and Biostatistics, Medical School Building, St Mary’s Hospital, Imperial College London, United Kingdom (V.K., M.-R.J., D.G.).; 7 Center for Life Course Health Research, Faculty of Medicine (V.K., M.-R.J.), University of Oulu, Finland.; 8 Research Unit of Mathematical Sciences (V.K.), University of Oulu, Finland.; 9 Biocenter Oulu (M.-R.J.), University of Oulu, Finland.; 10 MRC/Wits Developmental Pathways for Health Research Unit, Department of Pediatrics (T.C.), Faculty of Health Sciences, University of the Witwatersrand, Johannesburg, South Africa.; 11 Sydney Brenner Institute for Molecular Bioscience (T.C.), Faculty of Health Sciences, University of the Witwatersrand, Johannesburg, South Africa.; 12 The African Center of Excellence in Bioinformatics of Bamako (ACE-B), University of Sciences, Techniques and Technologies of Bamako, Mali (T.S., B.U.).; 13 Harvard Medical School, Boston, MA (I.D.).; 14 Clinical Pharmacology Group, Pharmacy and Medicines Directorate, St George’s University Hospitals NHS Foundation Trust, London, United Kingdom (D.K.R., A.T., D.G.).; 15 National Institute for Health Research, Cambridge Biomedical Research Centre, University of Cambridge and Cambridge University Hospitals, United Kingdom (A.M.M.).; 16 Corporal Michael J. Crescenz VA Medical Center, Philadelphia, PA (S.M.D., M.V.).; 17 Perelman School of Medicine, University of Pennsylvania, Philadelphia (S.M.D.).; 18 Department of Medicine, University of Pennsylvania Perelman School of Medicine, Philadelphia (M.V.).; 19 Department of Biology; Brody School of Medicine Center for Health Disparities, East Carolina University, Greenville, NC (K.L.K.).; 20 Brown Foundation Institute of Molecular Medicine, University of Texas Health Science Center at Houston (M.F.).; 21 Unit of Primary Care, Oulu University Hospital, Finland (M.-R.J.).; 22 Department of Life Sciences, College of Health and Life Sciences, Brunel University London, United Kingdom (M.-R.J.).; 23 Clinical Pharmacology and Therapeutics Section, Institute of Medical and Biomedical Education and Institute for Infection and Immunity, St George’s, University of London, United Kingdom (D.G.).; 24 Novo Nordisk Research Centre Oxford, Old Road Campus, United Kingdom (D.G.).

**Keywords:** cholesterol, ischemic stroke, lipid, mortality, risk factor

## Abstract

**Background and Purpose:**

Metabolic traits affect ischemic stroke (IS) risk, but the degree to which this varies across different ethnic ancestries is not known. Our aim was to apply Mendelian randomization to investigate the causal effects of type 2 diabetes (T2D) liability and lipid traits on IS risk in African ancestry individuals, and to compare them to estimates obtained in European ancestry individuals.

**Methods:**

For African ancestry individuals, genetic proxies for T2D liability and circulating lipids were obtained from a meta-analysis of the African Partnership for Chronic Disease Research study, the UK Biobank, and the Million Veteran Program (total N=77 061). Genetic association estimates for IS risk were obtained from the Consortium of Minority Population Genome-Wide Association Studies of Stroke (3734 cases and 18 317 controls). For European ancestry individuals, genetic proxies for the same metabolic traits were obtained from Million Veteran Program (lipids N=297 626, T2D N=148 726 cases, and 965 732 controls), and genetic association estimates for IS risk were obtained from the MEGASTROKE study (34 217 cases and 406 111 controls). Random-effects inverse-variance weighted Mendelian randomization was used as the main method, complemented with sensitivity analyses more robust to pleiotropy.

**Results:**

Higher genetically proxied T2D liability, LDL-C (low-density lipoprotein cholesterol), total cholesterol and lower genetically proxied HDL-C (high-density lipoprotein cholesterol) were associated with increased risk of IS in African ancestry individuals (odds ratio per doubling the odds of T2D liability [95% CI], 1.09 [1.07–1.11]; per standard-deviation increase in LDL-C, 1.12 [1.04–1.21]; total cholesterol: 1.23 [1.06–1.43]; HDL-C, 0.93 [0.89–0.99]). There was no evidence for differences in these estimates when performing analyses in European ancestry individuals.

**Conclusions:**

Our analyses support a causal effect of T2D liability and lipid traits on IS risk in African ancestry individuals, with Mendelian randomization estimates similar to those obtained in European ancestry individuals.

## Abstract 2

Supplemental Digital Content is available in the text.

Stroke is a major contributor to morbidity and mortality globally, responsible for over 5.5 million deaths per year.^1^ The global burden of stroke disproportionately affects low and middle-income countries, with over 85% of all stroke deaths occurring in these nations.^2^ Although stroke was historically seen as a disease affecting affluent regions, Africa now reports the highest incidence of stroke and the highest case fatality in the world.^3^ There is thus a growing need to understand the risk factors for stroke in African ancestry individuals.

Large multinational observational studies have established metabolic traits, such as dyslipidemia and type 2 diabetes (T2D), as risk factors for stroke.^4^ However, it is not clear how the effects of these risk factors vary between individuals of different genetic ancestries. Ethnic variation in stroke risk factors has previously been explored in observational studies, but these are liable to confounding and reverse causation, limiting the ability to make causal inferences.^5^

To address these issues, Mendelian randomization (MR) employs genetic variants as proxies for an exposure to study its effect on an outcome.^6^ MR is analogous to a randomized controlled trial with individuals being randomly assigned genetic variants at conception, minimizing confounding and reverse causality. MR has been widely used to examine risk factors for stroke in European populations.^7–9^ However, similar studies in other ethnic groups have not been undertaken, largely due to paucity of genetic data on individuals of non-European populations. The publication of the COMPASS (Consortium of Minority Population Genome-Wide Association Studies of Stroke)^10^ provides an opportunity to conduct MR studies in people of African ancestry.

Here, we used MR to investigate the causal effect of lipid traits and T2D liability on ischemic stroke (IS) risk in African ancestry populations and compared estimates to those obtained in individuals of European ancestry.

## Methods

### Ethical Approval, Data Availability, and Reporting

We used summary data from published studies that obtained relevant ethical approval and participant consent. These data are available on request to the original studies. The analysis codes are available on request to the corresponding author, and all results are presented in the main article or its Data Supplement.

### Genetic Association Estimates

We used 2-sample MR to investigate the associations of genetically proxied levels of five metabolic traits with IS risk: T2D liability, HDL-C (high-density lipoprotein cholesterol), LDL-C (low-density lipoprotein cholesterol), total cholesterol, and triglycerides. Genetic association estimates were obtained from publicly available summary statistics of genome-wide association studies (GWAS) detailed in Methods in the Data Supplement. Briefly, genetic associations for metabolic traits in African ancestry individuals were obtained from a meta-analysis of the African Partnership for Chronic Disease Research, self-reported Black participants in the UK Biobank, and African ancestry individuals in the Million Veteran Program, with a total of ≈77 000 participants. The genetic associations with the risk of IS were obtained from COMPASS, a GWAS meta-analysis of 3734 cases and 18 317 controls of African ancestry from 13 cohorts.^10^

For European ancestry individuals, genetic associations for the metabolic traits were obtained from the Million Veteran Program (T2D liability, 148 726 cases, 965 732 controls; lipids N=297 626) via database of Genotypes and Phenotypes.^11,12^ The genetic associations with the risk of IS were obtained from the MEGASTROKE consortium (34 217 cases, 406 111 controls, Methods in the Data Supplement).^13^ All genetic associations for both ancestries were adjusted for age, sex, and population stratification.

### Mendelian Randomization Analysis

For each exposure, we identified ancestry-specific instrumental variables for MR, based on GWAS on the exposure in the relevant ethnic group: variants that associated with the exposure at *P*<5×10^−8^ and were available in the outcome dataset were clumped at *r^2^*<0.01 within ±500 kb, using the corresponding reference ancestry in 1000 Genomes Project. The remaining variants were used as instrumental variables for MR.

To measure instrument strength, we calculated the variance explained and *F* statistics for the individual variants. To evaluate statistical power, we calculated the minimum detectable odds ratio (OR) for each exposure at power=0.8, given the exposure GWAS sample size, total variance explained by the genetic instruments (calculated as the sum of the variances explained by each individual instrument), and type I error rate=0.05.^14^

The main analyses estimating the association of genetically proxied levels of each exposure with risk of IS were performed using the random-effects inverse-variance weighted method.^15^ We examined the differences in the MR estimates between populations of European and African ancestries using the propagation of error method. Further sensitivity analyses—namely MR-Egger, weighted median, weighted mode, and contamination mixture method—were conducted to assess the robustness of the results to violations in instrumental variable assumptions (Methods in the Data Supplement).^15^ MR effect estimates are expressed as ORs per SD increase in genetically predicted levels of the exposure for continuous traits, and per doubling the odds (log-OR per unit change in exposure log-odds multiplied by log_e_[2]) in the exposure for T2D.

## Results

The demographics for African ancestry individuals in UK Biobank are given in Table 1, and the demographics for other considered populations can be found in their original publications.^10–13,16^ Table 2 provides minimum detectable ORs for MR analyses, and the association estimates for the variants used as instrumental variables and their individual *F* statistics are given in Table I in the Data Supplement.

**Table 1.**
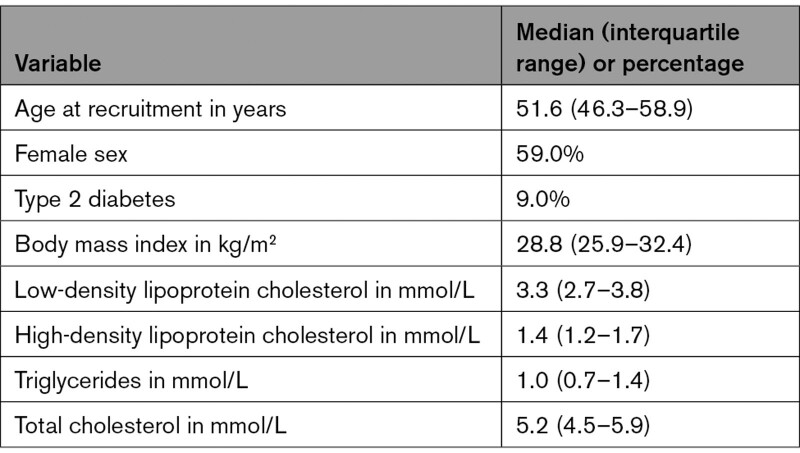
Demographics for African Ancestry Individuals in UKBiobank, N=6614

**Table 2.**
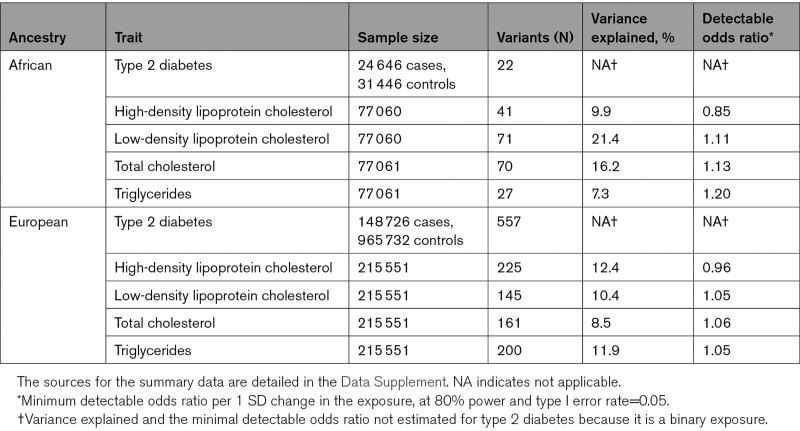
Exposure Summary Data and Statistical Power Calculations

In the MR analysis of African ancestry populations, higher genetically proxied T2D liability, LDL-C, and total cholesterol and lower genetically proxied HDL-C were associated with increased risk of IS (Figure; Table II in the Data Supplement). The MR estimate for triglycerides was similar in magnitude to the estimates for total cholesterol, LDL-C, and inversely to HDL-C, however with 95% CI for OR overlapping the null.

**Figure.**
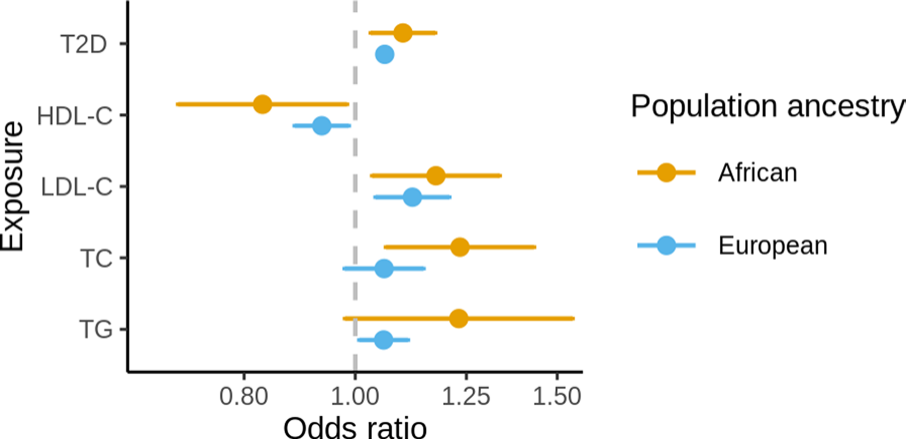
**Forest plot showing the main Mendelian randomization estimates for the association between genetically proxied lipid traits and type 2 diabetes (T2D) liability with risk of ischemic stroke in African and European ancestry populations.** Estimates represent odds ratios and their 95% CIs for ischemic stroke risk per 1 SD increase in genetically predicted levels of the exposure. HDL-C indicates high-density lipoprotein cholesterol; LDL-C, low-density lipoprotein cholesterol; TC, total cholesterol; and TG, triglycerides.

In European ancestry individuals, higher genetically proxied T2D liability, LDL-C, and lower genetically proxied HDL-C were associated with increased risk of IS (Figure; Table II in the Data Supplement). The effect estimate for genetically proxied total cholesterol was similar in the absolute value to other traits, however, with 95% CI for the OR marginally overlapping the null.

The comparison of MR estimates between European and African ancestry populations showed no strong evidence for differences in the MR estimates. The point estimates were marginally larger in African ancestry individuals for all traits (Table III in the Data Supplement). The associations between genetically proxied metabolic traits with the risk of IS were mostly consistent in the sensitivity analysis, apart from the estimates for HDL-C and triglycerides in European ancestry populations which were shrunk towards the null in the sensitivity analyses, implying some degree of horizontal pleiotropy (Table II and Figures I through VI in the Data Supplement).

## Discussion

This MR study found evidence for causal effects of lipid traits and T2D liability on IS risk for African ancestry individuals. When compared with the estimates obtained in European ancestry individuals, there was no evidence for marked differences in the effects.

These findings are of direct clinical relevance, as they support that optimization of these risk factors will be of benefit in reducing IS for all individuals, irrespective of ethnic background. However, although the effect of dyslipidemia and T2D on IS risk may be similar, it is also important to appreciate that the prevalence of these metabolic traits does vary considerably between different ethnic groups,^17^ resulting in marked differences in the proportion of stroke that can be attributed to these risk factors.^18^

By leveraging large-scale genetic association data from African and European populations, we were able to investigate the comparative effects of T2D liability and lipid traits on stroke risk in these ethnic groups. The use of genetically proxied metabolic traits in MR approach offers robustness against environmental confounding and reverse causation that can hinder causal inference in observational studies. The findings were mostly consistent in sensitivity analyses more robust to the inclusion of pleiotropic variants, suggesting that this is unlikely to be a major source of bias.

The limitations of this work should be acknowledged. The statistical power may have been insufficient to identify small differences in the MR estimates between European and African ancestry populations. The binary categorization of individuals as either of European or African ancestry is an over-simplification and will not capture the wider genetic diversity of individuals within each group. Furthermore, there may also be a population effect that impacts the genetic associations for individuals of the same ancestry when considered in different contexts.^19^ Genetic association estimates were pooled from studies of heterogeneous populations with varying demographics. Despite the adjustments for age, sex, and population stratification, population heterogeneity may introduce bias to the MR estimates. Summary statistics for the Million Veteran Program data were publicly available via database of Genotypes and Phenotypes only for variants with *P*<10^-4^, and therefore, we were not able to conduct multivariable MR to investigate the mutually adjusted, direct effect of each considered cardiometabolic trait. Similarly, nor could we perform bidirectional MR to explore for reverse causality. We could not expand our analyses to other cardiometabolic traits, such as blood pressure or obesity, as sufficiently large GWAS summary statistics on these traits in African ancestry populations were not available to us. Finally, we were not able to examine the associations across different stroke subtypes, as subtype-specific GWAS summary statistics were not available in COMPASS.

In conclusion, our results are consistent with T2D liability and lipid traits having a similar effect on IS risk in both African and European ancestry populations. Optimization of these risk factors will be of benefit for reducing the population burden of IS.

## Acknowledgments

The COMPASS (Consortium of Minority Population Genome-Wide Association Studies of Stroke) project would like to thank the staff and participants of the ARIC (Atherosclerosis Risk in Communities), CHS (Cardiovascular Health Study), VISP (Vitamin Intervention for Stroke Prevention), HANDLS (Healthy Aging in Neighborhoods of Diversity Across the Life Span), INTERSTROKE, ISGS (Ischemic Stroke Genetics Study), JHS (Jackson Heart Study), SIGNET-REGARDS (Sea Islands Genetics Network–Reasons for Geographic and Racial Differences in Stroke), GEOS (Genetics of Early Onset Stroke), SiGN (Stroke Genetics Network), SLESS (South London Ethnicity and Stroke Study), SWISS (Siblings With Ischemic Stroke Study), and WHI (Women’s Health Initiative) studies for their dedication, and willingness to participate in the respective research studies, which made this work possible. The MEGASTROKE project received funding from sources specified at http://www.megastroke.org/acknowledgments.html. Drs Fatumo and Gill designed the study. Drs Karhunen and Fatumo performed statistical analyses. Drs Fatumo, Karhunen, D.K. Ryan, A. Taylor, Dr Gill drafted the article. All authors interpreted results, edited the article for intellectual content, and take responsibility for the integrity of the study. Drs Fatumo and Karhunen had full access to all the data in the study and take responsibility for the integrity of the data and the accuracy of the data analysis.

## Sources of Funding

Dr Fatumo is funded by the Wellcome International Intermediate fellowship (220740/Z/20/Z) at the MRC/UVRI and LSHTM. Dr Gill is supported by the Wellcome 4i Program (203928/Z/16/Z) and by a National Institute for Health Research Clinical Lectureship (CL-2020-16-001) at St. George’s, University of London. Drs Karhunen and Gill are supported by British Heart Foundation Centre of Research Excellence (RE/18/4/34215) at Imperial College London. Dr Chikowore is an international training fellow supported by the Wellcome Trust grant (214205/Z/18/Z). B. Udosen and M. Nakabuye are supported by the Wellcome Trust. M. Nakabuye and S.F. received support from the Makerere University-Uganda Virus Research Institute Centre of Excellence for Infection and Immunity Research and Training (MUII). MUII is supported through The Developing Excellence in Leadership, Training and Science (DELTAS) Africa Initiative (grant 107743). The DELTAS Africa Initiative is an independent funding scheme of the African Academy of Sciences (AAS), Alliance for Accelerating Excellence in Science in Africa (AESA), and supported by the New Partnership for Africa’s Development Planning and Coordinating Agency (NEPAD Agency) with funding from the Wellcome Trust (107743) and the UK government. M. Nakabuye acknowledges the support of Makerere University Non-Communicable Diseases (MacNCD). This research is made possible by the MakNCD Research Training Program: National Institutes of Health (NIH) 1D43TW011401-01 through the Fogarty International Center (FIC). CE, B. Udosen, and Dr Fatumo acknowledge H3Africa Bioinformatics Network (H3ABioNet) Node, National Biotechnology Development Agency (NABDA), and the Center for Genomics Research and Innovation (CGRI) Abuja, Nigeria. Dr Damrauer was supported by the Department of Veterans Affairs Office of Research and Development (IK2-CX001780). Dr Mason is funded by the EC-Innovative Medicines Initiative (BigData@Heart). Dr Burgess is supported by a Sir Henry Dale Fellowship jointly funded by the Wellcome Trust and the Royal Society (204623/Z/16/Z). This research was funded by UK Research and Innovation (UKRI) Medical Research Council (MC_UU_00002/7) and supported by the National Institute for Health Research (NIHR) Cambridge Biomedical Research Centre (BRC-1215-20014). The views expressed are those of the author(s) and not necessarily those of the NIHR or the Department of Health and Social Care. The funding sources did not have any role in designing the study, performing analysis, or communicating findings.

## Disclosures

Dr Damrauer has received grants from the US Department of Veterans Affairs, Calico Labs, and Renalytix AI plc outside the submitted work. Dr Gill is employed part-time by Novo Nordisk. T. Sounkou reports grants and personal fees from National Institutes of Health (NIH) Cooperative Agreement 1 U2R TW010673-01 for West African Center of Excellence for Global Health Bioinformatics Research Training during the conduct of the study; personal fees from NIH Cooperative Agreement 1 U2R TW010673-01 for West African Center of Excellence for Global Health Bioinformatics Research Training outside the submitted work. Dr Mason reports grants from National Institute for Health Research (Cambridge Biomedical Research Centre at the Cambridge University Hospitals National Health Service [NHS] Foundation Trust) and grants from EC-Innovative Medicines Initiative (BigData@Heart) during the conduct of the study. The other authors report no conflicts.

## Supplemental Materials

Expanded Methods

Online Tables I–III

Online Figures I–VI

References 20–22

## Supplementary Material



## References

[1] Donkor ES. Stroke in the 21^st^ century: a snapshot of the burden, epidemiology, and quality of life. Stroke Res Treat. 2018; 2018:3238165. doi: 10.1155/2018/3238165 30598741 10.1155/2018/3238165 PMC6288566

[2] Owolabi M Akarolo-Anthony S Akinyemi R Arnett D Gebregziabher M Jenkins C Tiwari H Arulogun O Akpalu A Sarfo F. The burden of stroke in Africa: a glance at the present and a glimpse into the future: review article. CVJA. 2015; 26:S27–S38 25962945 10.5830/CVJA-2015-038 PMC4557491

[3] Owolabi M Olowoyo P Popoola F Lackland D Jenkins C Arulogun O Akinyemi R Akinyemi O Akpa O Olaniyan O. The epidemiology of stroke in Africa: a systematic review of existing methods and new approaches. J Clin Hypertens (Greenwich). 2018; 20:47–55. doi: 10.1111/jch.13152 29228472 10.1111/jch.13152 PMC5777902

[4] O’Donnell MJ Chin SL Rangarajan S Xavier D Liu L Zhang H Rao-Melacini P Zhang X Pais P Agapay S; INTERSTROKE Investigators. Global and regional effects of potentially modifiable risk factors associated with acute stroke in 32 countries (INTERSTROKE): a case-control study. Lancet. 2016; 388:761–775. doi: 10.1016/S0140-6736(16)30506-2 27431356 10.1016/S0140-6736(16)30506-2

[5] Agyemang C Addo J Bhopal R Aikins Ade G Stronks K. Cardiovascular disease, diabetes and established risk factors among populations of sub-Saharan African descent in Europe: a literature review. Global Health. 2009; 5:7. doi: 10.1186/1744-8603-5-7 19671137 10.1186/1744-8603-5-7 PMC2734536

[6] Davies NM Holmes MV Davey Smith G. Reading Mendelian randomisation studies: a guide, glossary, and checklist for clinicians. BMJ. 2018; 362:k601. doi: 10.1136/bmj.k601 30002074 10.1136/bmj.k601 PMC6041728

[7] Hopewell JC Clarke R. Emerging risk factors for stroke: what have we learned from Mendelian randomization studies? Stroke. 2016; 47:1673–1678. doi: 10.1161/STROKEAHA.115.010646 27094995 10.1161/STROKEAHA.115.010646

[8] Larsson SC Scott RA Traylor M Langenberg CC Hindy G Melander O Orho-Melander M Seshadri S Wareham NJ Markus HS; METASTROKE Collaboration and NINDS Stroke Genetics Network (SiGN). Type 2 diabetes, glucose, insulin, BMI, and ischemic stroke subtypes: Mendelian randomization study. Neurology. 2017; 89:454–460. doi: 10.1212/WNL.0000000000004173 28667182 10.1212/WNL.0000000000004173 PMC5539736

[9] Hindy G Engström G Larsson SC Traylor M Markus HS Melander O Orho-Melander M; Stroke Genetics Network (SiGN). Role of blood lipids in the development of ischemic stroke and its subtypes: a Mendelian Randomization Study. Stroke. 2018; 49:820–827. doi: 10.1161/STROKEAHA.117.019653 29535274 10.1161/STROKEAHA.117.019653 PMC5895121

[10] Keene KL Hyacinth HI Bis JC Kittner SJ Mitchell BD Cheng YC Pare G Chong M O’Donnell M Meschia JF; COMPASS, SiGN, and METASTROKE Consortia. Genome-wide association study meta-analysis of stroke in 22 000 individuals of African descent identifies novel associations with stroke. Stroke. 2020; 51:2454–2463. doi: 10.1161/STROKEAHA.120.029123 32693751 10.1161/STROKEAHA.120.029123 PMC7387190

[11] Klarin D Damrauer SM Cho K Sun YV Teslovich TM Honerlaw J Gagnon DR DuVall SL Li J Peloso GM; Global Lipids Genetics Consortium, Myocardial Infarction Genetics (MIGen) Consortium, The Geisinger-Regeneron DiscovEHR Collaboration, The VA Million Veteran Program. Genetics of blood lipids among ~300,000 multi-ethnic participants of the Million Veteran Program. Nat Genet. 2018; 50:1514–1523. doi: 10.1038/s41588-018-0222-9 30275531 10.1038/s41588-018-0222-9 PMC6521726

[12] Vujkovic M Keaton JM Lynch JA Miller DR Zhou J Tcheandjieu C Huffman JE Assimes TL Lorenz K Zhu X; HPAP Consortium; Regeneron Genetics Center; VA Million Veteran Program. Discovery of 318 new risk loci for type 2 diabetes and related vascular outcomes among 1.4 million participants in a multi-ancestry meta-analysis. Nat Genet. 2020; 52:680–691. doi: 10.1038/s41588-020-0637-y 32541925 10.1038/s41588-020-0637-y PMC7343592

[13] Malik R Chauhan G Traylor M Sargurupremraj M Okada Y Mishra A Rutten-Jacobs L Giese AK van der Laan SW Gretarsdottir S; AFGen Consortium; Cohorts for Heart and Aging Research in Genomic Epidemiology (CHARGE) Consortium; International Genomics of Blood Pressure (iGEN-BP) Consortium; INVENT Consortium; STARNET; BioBank Japan Cooperative Hospital Group; COMPASS Consortium; EPIC-CVD Consortium; EPIC-InterAct Consortium; International Stroke Genetics Consortium (ISGC); METASTROKE Consortium; Neurology Working Group of the CHARGE Consortium; NINDS Stroke Genetics Network (SiGN); UK Young Lacunar DNA Study; MEGASTROKE Consortium. Multiancestry genome-wide association study of 520,000 subjects identifies 32 loci associated with stroke and stroke subtypes. Nat Genet. 2018; 50:524–537. doi: 10.1038/s41588-018-0058-329531354

[14] Burgess S. Sample size and power calculations in Mendelian randomization with a single instrumental variable and a binary outcome. Int J Epidemiol. 2014; 43:922–929. doi: 10.1093/ije/dyu005 24608958 10.1093/ije/dyu005 PMC4052137

[15] Slob EAW Burgess S. A comparison of robust Mendelian randomization methods using summary data. Genet Epidemiol. 2020; 44:313–329. doi: 10.1002/gepi.22295 32249995 10.1002/gepi.22295 PMC7317850

[16] Gurdasani D Carstensen T Fatumo S Chen G Franklin CS Prado-Martinez J Bouman H Abascal F Haber M Tachmazidou I. Uganda genome resource enables insights into population history and genomic discovery in Africa. Cell. 2019; 179:984–1002.e36. doi: 10.1016/j.cell.2019.10.004 31675503 10.1016/j.cell.2019.10.004 PMC7202134

[17] Cruz-Flores S Rabinstein A Biller J Elkind MS Griffith P Gorelick PB Howard G Leira EC Morgenstern LB Ovbiagele B; American Heart Association Stroke Council; Council on Cardiovascular Nursing; Council on Epidemiology and Prevention; Council on Quality of Care and Outcomes Research. Racial-ethnic disparities in stroke care: the American experience: a statement for healthcare professionals from the American Heart Association/American Stroke Association. Stroke. 2011; 42:2091–2116. doi: 10.1161/STR.0b013e3182213e24 21617147 10.1161/STR.0b013e3182213e24

[18] Hajat C Tilling K Stewart JA Lemic-Stojcevic N Wolfe CD. Ethnic differences in risk factors for ischemic stroke: a European case-control study. Stroke. 2004; 35:1562–1567. doi: 10.1161/01.STR.0000131903.04708.b8 15192251 10.1161/01.STR.0000131903.04708.b8

[19] Gola D Erdmann J Läll K Mägi R Müller-Myhsok B Schunkert H König IR. Population Bias in polygenic risk prediction models for coronary artery disease. Circ Genom Precis Med. 2020; 13:e002932. doi: 10.1161/CIRCGEN.120.002932 33170024 10.1161/CIRCGEN.120.002932

[20] Bycroft C Freeman C Petkova D Band G Elliott LT Sharp K Motyer A Vukcevic D Delaneau O O’Connell J. The UK Biobank resource with deep phenotyping and genomic data. Nature. 2018; 562:203–209. doi: 10.1038/s41586-018-0579-z 30305743 10.1038/s41586-018-0579-z PMC6786975

[21] Gaziano JM Concato J Brophy M Fiore L Pyarajan S Breeling J Whitbourne S Deen J Shannon C Humphries D. Million Veteran Program: a mega-biobank to study genetic influences on health and disease. J Clin Epidemiol. 2016; 70:214–223. doi: 10.1016/j.jclinepi.2015.09.016 26441289 10.1016/j.jclinepi.2015.09.016

[22] Wasserstein RL Schirm AL Lazar NA. Moving to a World Beyond “p < 0.05.” Am Stat. 2019; 73:1–19

