# Supplementary material for: Metabolic Traits and Stroke Risk in Individuals of African Ancestry: Mendelian Randomization Analysis
Source: Stroke. 2021 Jun 3;52(8):2680–4. doi: 10.1161/STROKEAHA.121.034747 (PMC8312569; doi:10.1161/STROKEAHA.121.034747)
Supplement: Supplementary file 1 [file str-52-2680-s001.pdf]

SUPPLEMENTARY MATERIAL

Metabolic traits and stroke risk in individuals of African ancestry: Mendelian randomization analysis

TABLE OF CONTENTS

SUPPLEMENTARY METHODS **2**

GENETIC ASSOCIATION ESTIMATES 2

MENDELIAN RANDOMIZATION 3

MEASURING STATISTICAL EVIDENCE 3

SUPPLEMENTARY TABLE I **4**

SUPPLEMENTARY TABLE II **5**

SUPPLEMENTARY TABLE III **6**

SUPPLEMENTARY FIGURE I **7**

SUPPLEMENTARY FIGURE II **8**

SUPPLEMENTARY FIGURE III **9**

SUPPLEMENTARY FIGURE IV **10**

SUPPLEMENTARY FIGURE V **11**

SUPPLEMENTARY FIGURE VI **12**

SUPPLEMENTARY METHODS

GENETIC ASSOCIAITON ESTIMATES

AFRICAN ANCESTRY INDIVIDUALS

For the metabolic traits used as exposures, genetic association estimates for populations of African ancestries were obtained from a genome-wide association study (GWAS) meta-analysis of the African Partnership for Chronic Disease Research (APCDR, N=13,612), Black participants in the UKBiobank (N=6,614) and African ancestry individuals from in the Million Veteran Program (MVP, N=57,332). APCDR data used here consists of four cohorts from Sub-Saharan Africa: The Uganda Genome Resource (UGR), The Durban Diabetes Study (DDS), The Durban Case Control Study (DCC), The Africa America Diabetes Mellitus Study (AADM)[16]. UKBiobank is a prospective study of approximately 500,000 UK residents[20], and MVP is an observational cohort study of US military force veterans[21].

We used GWAS summary data from Consortium of Minority Population Genome-Wide Association Studies of Stroke (COMPASS) to obtain genetic association estimates with the risk of ischemic stroke (IS)[10]. COMPASS comprises a total of 22,051 individuals of African descent with either a physician-adjudicated stroke (N=3,734) or no history of stroke (N=18,317). This consortium includes participants from 13 cohorts which include: prospective cohorts (ARIC study [Atherosclerosis Risk in Communities], CHS [Cardiovascular Health Study], JHS [Jackson Heart Study], the WHI [Women's Health Initiative]; case-control studies (INTERSTROKE, REGARDS [Reasons for Geographic and Racial Differences in Stroke], ISGS [Ischemic Stroke Genetics Study], VISP [Vitamin Intervention for Stroke Prevention], SLESS [South London Ethnicity and Stroke Study], the GEOS Study [Genetics of Early Onset Stroke], the NINDS-SiGN [National Institute of Neurological Disorders and Stroke–Stroke Genetics Network], HANDLS [Healthy Aging in Neighborhoods of Diversity Across the Life Span]); and an affected sib pair study—SWISS (Siblings With Ischemic Stroke Study). This consortium defined stroke as a focal neurological deficit of presumed vascular cause with a sudden onset and lasting 24 hours or until death with clinical or radiological (computed tomography/magnetic resonance imaging) evidence with stroke diagnosis made when there is overwhelming clinical evidence in the absence of radiological evidence of a cerebral infarction. They only considered first (incident) clinically validated ischemic strokes. Individuals with a baseline history of ischemic or hemorrhagic stroke were excluded[10].

EUROPEAN ANCESTRY INDIVIDUALS

We obtained genetic associations with the risk of IS in European ancestry individuals from the MEGASTROKE consortium (34,217 cases, 406,111 controls)[13]. This includes cohorts from the Australian Stroke Genetics Collaborative (ASGC), Bio-Repository of DNA in Stroke (BRAINS), Genetics of Early Onset Stroke (GEOS) Study, Heart Protection Study (HPS), Ischemic Stroke Genetics Study (ISGS)/ Siblings With Ischemic Stroke Study (SWISS), MGH Genes Affecting Stroke Risk and Outcome Study (MGH-GASROS), Milano, Wellcome Trust Case-Control Consortium 2 (WTCCC2), VISP, WHI - The Women's Health Initiative Hormone Trial HT. Strokes were defined as IS based on clinical and imaging criteria.

MENDELIAN RANDOMIZATION

The main Mendelian randomization (MR) analyses estimating the association of genetically proxied levels of each exposure with risk of IS were performed using the random-effects inverse-variance weighted (IVW) method[15]. The IVW method combines the effect estimates from each individual genetic variant (computed as the ratio of the variant-IS association to the variant-exposure association). We also examined the evidence for differences in the MR estimates between populations of European and African ancestries. The standard error (SE) for the difference was estimated using the propagation of error method:

$$SE(\beta_{EUR} - \beta_{AFR}) = \sqrt{SE(\beta_{EUR})^2 + SE(\beta_{AFR})^2},$$

where β_{EUR} and β_{AFR} are the MR estimates in European and African ancestry populations, respectively.

IVW method provides a statistically consistent estimator of the true causal effect as long as all genetic variants are valid instrumental variables, fulfilling the following three assumptions: First, the genetic instruments are strongly predictive of cardiometabolic disorder. Second, the association of genetic instruments with the risk of IS is not confounded. Last, the effect of the genetic instrument on the risk of IS should be fully mediated via cardiometabolic disorder under investigation[15]. If any of the genetic variants are invalid instruments due to pleiotropy (where the variant affects IS risk through pathways unrelated to the exposure), the IVW estimator is still statistically consistent as long as the pleiotropy is balanced (that is, the average of the pleiotropic effects of each genetic variant on the outcome are equal to zero).

Sensitivity analyses for MR were performed using the weighted median method, a weighted mode-based method, the contamination mixture method, and the MR-Egger method[15]. Each of these methods provides a statistically consistent estimate of the true causal effect under different sets of assumptions. The MR-Egger method allows all genetic variants to be invalid but requires the pleiotropic effects that relate to the risk of IS through pleiotropic pathways to be uncorrelated with the genetic variant-exposure associations. Weighted median provides a consistent estimate if at least half of the weights are provided by valid instrumental variables. Both weighted mode and contamination mixture method rely on the zero modal pleiotropy assumption, i.e. the largest weights among different homogeneous subsets should be provided by valid instrumental variables.

MEASURING STATISTICAL EVIDENCE

To guide the interpretation of the results, a Bonferroni-corrected significance level for five exposures is 0.05/5 = 0.01. However, the statistical evidence is interpreted in light of the effect sizes, their confidence intervals, and the consistency of the results in the sensitivity analyses, and we refrain from categorizing results as ‘significant’ or ‘non-significant’ based on a single p-value threshold[22].

Supplementary Table I. Genetic variants used as instrumental variables for each exposure. T2DM: type 2 diabetes mellitus; HDL: high-density lipoprotein, LDL: low-density lipoprotein; TC: Total cholesterol; TG: triglycerides; AFR: association estimates in populations of African ancestries; EUR: association estimates in populations of European ancestries; CHR: chromosome; POS: position; EA: effect allele; NEA: non-effect allele. See separate Excel file.

Supplementary Table II. Mendelian Randomization odds ratios (OR) and 95% confidence intervals (CI) on the risk of stroke per one standard deviation increase in the exposure, using different MR methods. Con-Mix: Contamination mixture; IVW: random-effects Inverse-variance weighted; HDL: high-density lipoprotein, LDL: low-density lipoprotein; TG: triglycerides; TC: Total cholesterol; Median: weighted median-based estimate; Mode: weighted mode-based estimate; MR-Egger: Mendelian Randomization Egger; T2DM: type 2 diabetes mellitus. See the separate Excel file.

Supplementary Table III. Comparison of Mendelian Randomization odds ratios (OR) and their 95% confidence intervals (CI) per standard deviation increase in the exposure (per doubling the odds of type 2 diabetes mellitus [T2DM]) on the risk of ischemic stroke between populations of African and European ancestries. HDL-C: high-density lipoprotein cholesterol, LDL-C: low-density lipoprotein cholesterol; TC: Total cholesterol; TG: triglycerides.

	**African**	**European**	
**Exposure**	**OR (95% CI)**	**OR (95% CI)**	**P-value for difference**
T2DM	1.15 (1.05 – 1.26)	1.09 (1.07 – 1.11)	0.28
HDL-C	0.83 (0.70 – 0.98)	0.93 (0.89 – 0.99)	0.19
LDL-C	1.18 (1.03 – 1.34)	1.12 (1.04 – 1.21)	0.53
TC	1.23 (1.06 – 1.43)	1.06 (0.98 – 1.15)	0.08
TG	1.23 (0.98 – 1.55)	1.06 (1.01 – 1.11)	0.21

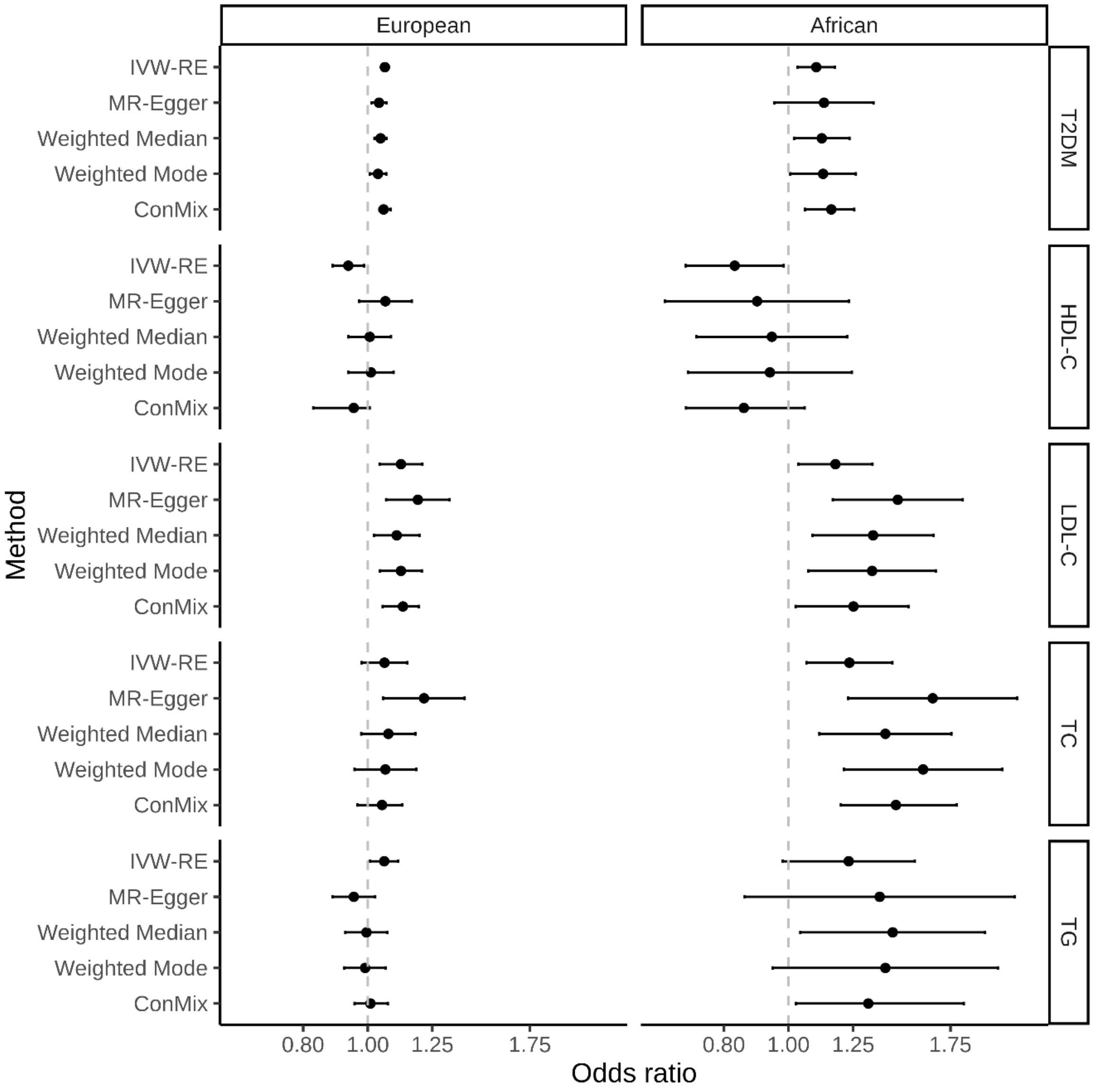

Supplementary Figure I: Forest plot showing Mendelian randomization sensitivity analysis estimates for the association between cardiometabolic traits and risk of ischemic stroke. Results are given as point estimates and their 95% confidence intervals per standard deviation increase in genetically proxied levels of the exposure for continuous traits and per doubling of the odds for genetically proxied type 2 diabetes mellitus (T2DM) liability. HDL-C: high-density lipoprotein cholesterol, LDL-C: low-density lipoprotein cholesterol; TC: Total cholesterol; TG: triglycerides; IVW-RE: random-effects inverse-variance weighted; MR-Egger: Mendelian randomization Egger; Con-Mix: Contamination mixture.

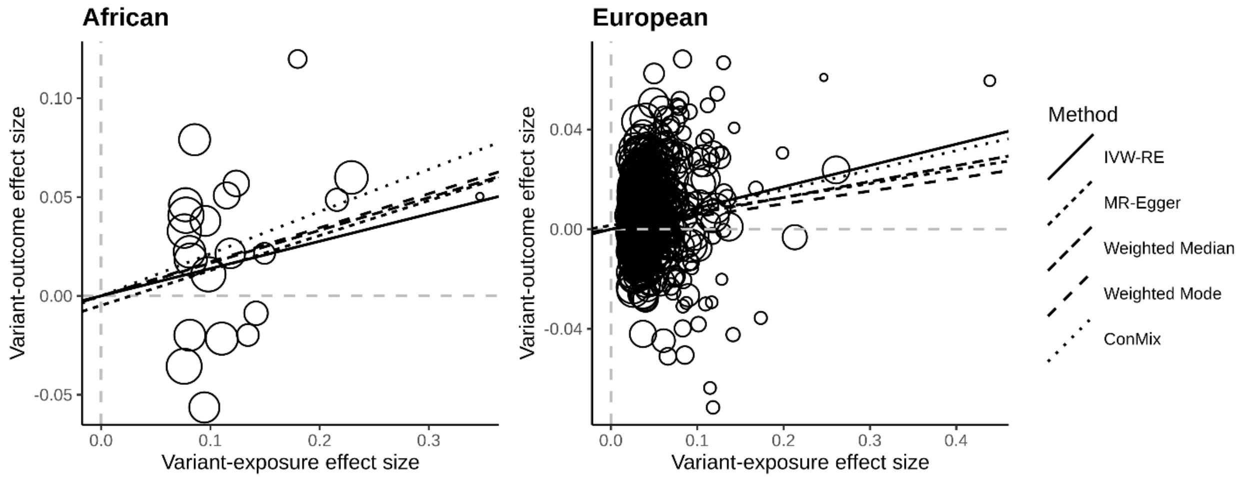

Supplementary Figure II. A scatterplot of instrumental variable genetic association estimates for type 2 diabetes risk (log-odds, x-axis) and ischemic stroke risk (log-odds, y-axis) in Mendelian randomization (MR) analysis, separately for African and European ancestries, with lines added for MR results using different methods. The slope of each line corresponds to the effect size obtained by the corresponding MR method. The size of each data point corresponds to the precision of the variant-outcome effect size estimate. IVW-RE: random-effects Inverse-variance weighted; MR-Egger: Mendelian randomization Egger; ConMix: contamination mixture method.

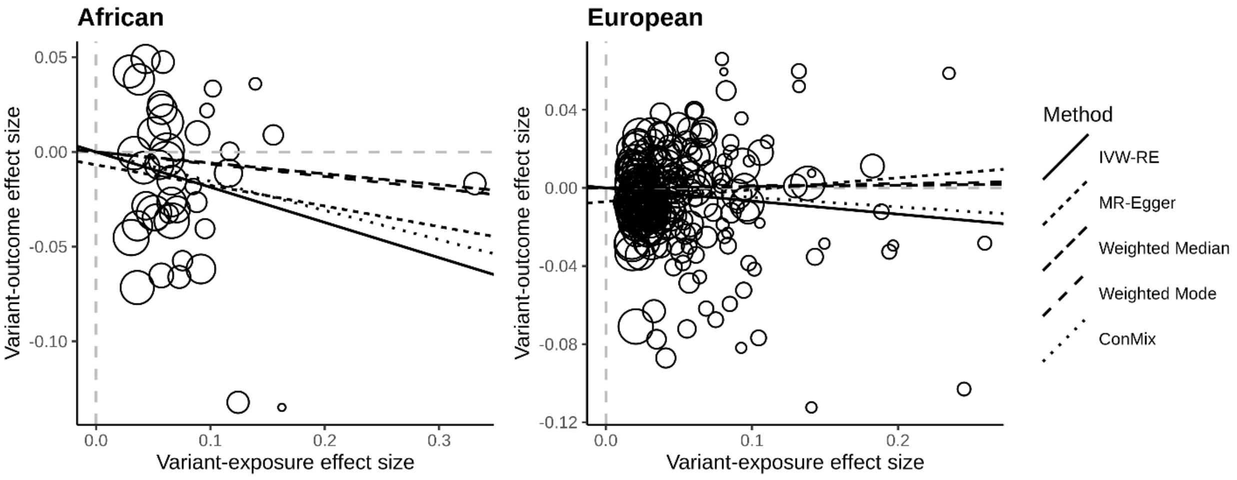

Supplementary Figure III. A scatterplot of instrumental variable genetic association estimates for high-density lipoprotein cholesterol (x-axis) and ischemic stroke risk (log-odds, y-axis) in Mendelian randomization (MR) analysis, separately for African and European ancestries, with lines added for MR results using different methods. The slope of each line corresponds to the effect size obtained by the corresponding MR method. The size of each data point corresponds to the precision of the variant-outcome effect size estimate. IVW-RE: random-effects Inverse-variance weighted; MR-Egger: Mendelian randomization Egger; ConMix: contamination mixture method.

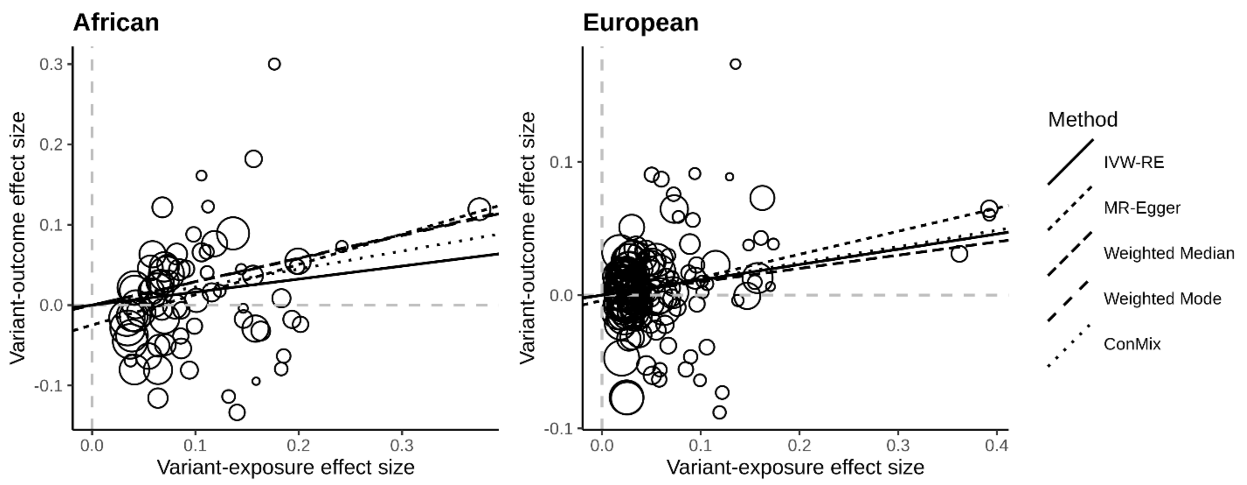

Supplementary Figure IV. A scatterplot of instrumental variable genetic association estimates for low-density lipoprotein cholesterol (x-axis) and ischemic stroke risk (log-odds, y-axis) in Mendelian randomization (MR) analysis, separately for African and European ancestries, with lines added for MR results using different methods. The slope of each line corresponds to the effect size obtained by the corresponding MR method. The size of each data point corresponds to the precision of the variant-outcome effect size estimate. IVW-RE: random-effects Inverse-variance weighted; MR-Egger: Mendelian randomization Egger; ConMix: contamination mixture method.

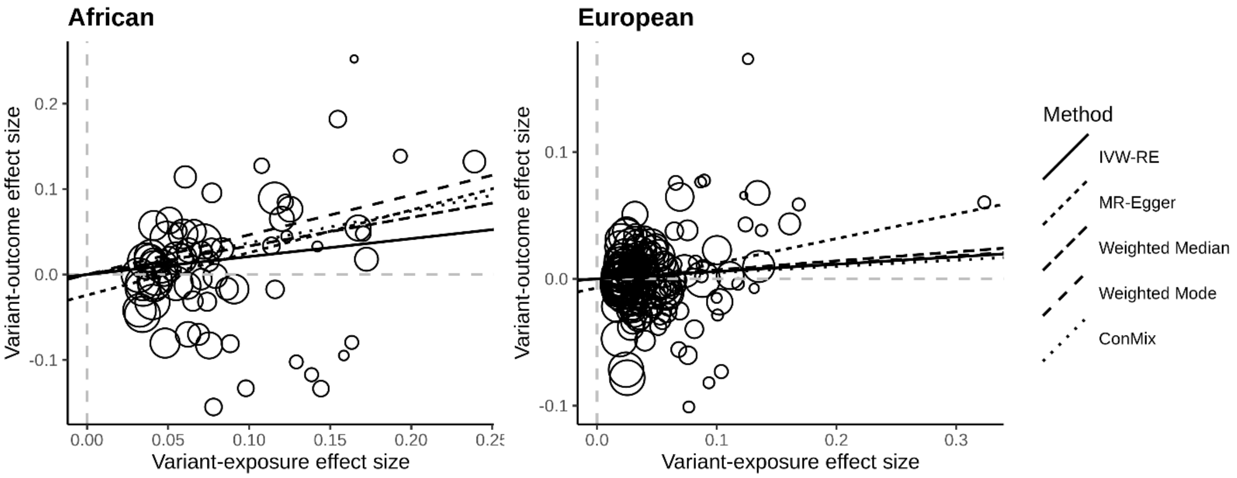

Supplementary Figure V. A scatterplot of instrumental variable genetic association estimates for total cholesterol (x-axis) and ischemic stroke risk (log-odds, y-axis) in Mendelian randomization (MR) analysis, separately for African and European ancestries, with lines added for MR results using different methods. The slope of each line corresponds to the effect size obtained by the corresponding MR method. The size of each data point corresponds to the precision of the variant-outcome effect size estimate. IVW-RE: random-effects Inverse-variance weighted; MR-Egger: Mendelian randomization Egger; ConMix: contamination mixture method.

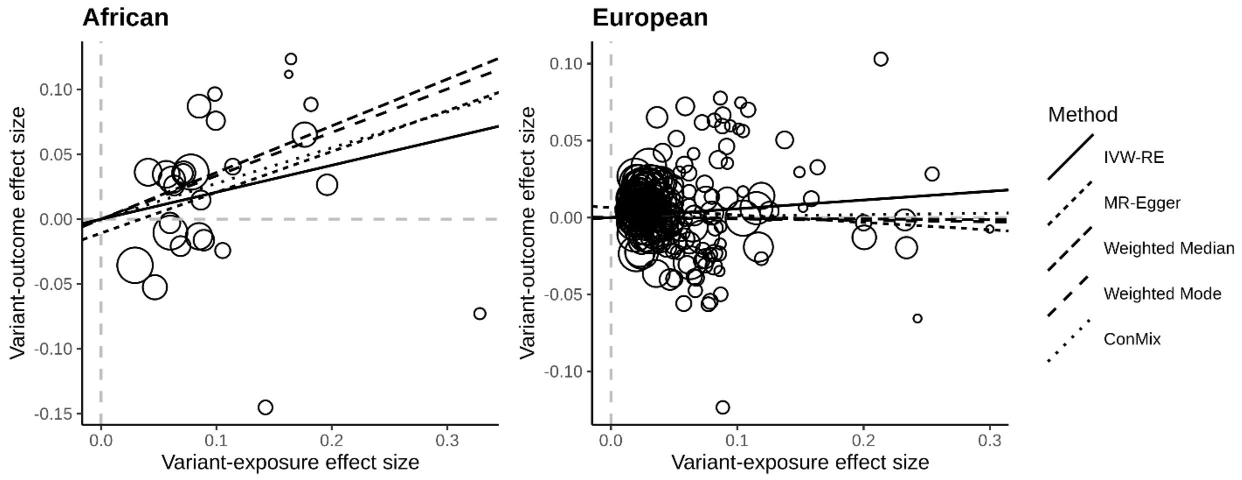

Supplementary Figure VI. A scatterplot of instrumental variable genetic association estimates for triglycerides (x-axis) and ischemic stroke risk (log-odds, y-axis) in Mendelian randomization (MR) analysis, separately for African and European ancestries, with lines added for MR results using different methods. The slope of each line corresponds to the effect size obtained by the corresponding MR method. The size of each data point corresponds to the precision of the variant-outcome effect size estimate. IVW-RE: random-effects Inverse-variance weighted; MR-Egger: Mendelian randomization Egger; ConMix: contamination mixture method.
