# Supplementary material for: Metabolic Traits and Stroke Risk in Individuals of African Ancestry: Mendelian Randomization Analysis
Source: Stroke. 2021 Jun 3;52(8):2680–4. doi: 10.1161/STROKEAHA.121.034747 (PMC8312569; doi:10.1161/STROKEAHA.121.034747)
Supplement: Supplementary file 5 [file str-52-2680-s005.pdf]

Change of Authorship Form

(Must be completed and signed by ALL authors)

Please check all that apply

✔ New author(s) have been added (in addition to this form, all new authors must complete the copyright transfer agreement and conflict of interest disclosure.

☐ Change in order of authorship.

☐ An author wishes to remove his/her name. An author's name may only be removed his/her own request and a letter signed by the author should accompany this form

Manuscript Number STROKE/2020/034093

Manuscript Title Metabolic traits and stroke risk in individuals of African ancestry: Mendelian randomization analysis

Former Authorship

Please list ALL AUTHORS in the same order as the original submission. For more than 12, use an extra sheet.

**Print Name**		**Print Name**	
Name (1)	Scott M. Damrauer	Name (7)	Dipender Gill
Name (2)	Marijana Vujkovic	Name (8)	
Name (3)	Keith L. Keene	Name (9)	
Name (4)	Myriam Fornage	Name (10)	
Name (5)	Marjo-Riitta Järvelin	Name (11)	
Name (6)	Stephen Burgess	Name (12)	

New Authorship

All authors must sign below agreeing to the changes in authorship. The authorship order must reflect the authorship order of the manuscript.

Name		Signature	Date
Name (1)	Scott M. Damrauer		
Name (2)	Marijana Vujkovic		
Name (3)	Keith L. Keene		
Name (4)	Myriam Fornage		
Name (5)	Marjo-Riitta Järvelin		
Name (6)	Stephen Burgess		
Name (7)	Dipender Gill	[signature]	2021-02-18
Name (8)			
Name (9)			
Name (10)			
Name (11)			
Name (12)			

Please scan and email to stroke@strokeahajournal.org.
