# Supplementary material for: Metabolic Traits and Stroke Risk in Individuals of African Ancestry: Mendelian Randomization Analysis
Source: Stroke. 2021 Jun 3;52(8):2680–4. doi: 10.1161/STROKEAHA.121.034747 (PMC8312569; doi:10.1161/STROKEAHA.121.034747)
Supplement: Supplementary file 7 [file str-52-2680-s007.pdf]

Change of Authorship Form

(Must be completed and signed by ALL authors)

Please check all that apply

_✓_ New author(s) have been added (in addition to this form, all new authors must complete the copyright transfer agreement and conflict of interest disclosure.

_____ Change in order of authorship.

_____ An author wishes to remove his/her name. An author's name may only be removed his/her own request and a letter signed by the author should accompany this form

Manuscript Number STROKE/2020/034093

Manuscript Title Metabolic traits and stroke risk in individuals of African ancestry: Mendelian randomization analysis

Former Authorship

Please list ALL AUTHORS in the same order as the original submission. For more than 12, use an extra sheet.

Print Name	Print Name
Name (1) Segun Fatumo	Name (7) Mariam Nakabuye
Name (2) Ville Karhunen	Name (8) Opeyemi Soremekun
Name (3) Tinashe Chikowore	Name (9) Iyas Daghlas
Name (4) Toure Sounkou	Name (10) David K. Ryan
Name (5) Brenda Udosen	Name (11) Amybel Taylor
Name (6) Chisom Ezenwa	Name (12)

New Authorship

All authors must sign below agreeing to the changes in authorship. The authorship order must reflect the authorship order of the manuscript.

Name	Signature	Date
Name (1) Segun Fatumo	Signature	Date
Name (2) Ville Karhunen	Signature	Date
Name (3) Tinashe Chikowore	Signature	Date
Name (4) Toure Sounkou	Signature	Date
Name (5) Brenda Udosen	Signature	Date
Name (6) Chisom Ezenwa	Signature	Date
Name (7) Mariam Nakabuye	Signature	Date
Name (8) Opeyemi Soremekun	Signature	Date
Name (9) Iyas Daghlas	Signature	Date
Name (10) David K. Ryan	Signature	Date
Name (11) Amybel Taylor	Signature Amybel Taylor	Date 12-2-21
Name (12) Amy M. Mason	Signature	Date

Please scan and email to stroke@strokeahajournal.org.
