# Supplementary material for: Metabolic Traits and Stroke Risk in Individuals of African Ancestry: Mendelian Randomization Analysis
Source: Stroke. 2021 Jun 3;52(8):2680–4. doi: 10.1161/STROKEAHA.121.034747 (PMC8312569; doi:10.1161/STROKEAHA.121.034747)
Supplement: Supplementary file 8 [file str-52-2680-s008.pdf]

Change of Authorship Form

(Must be completed and signed by ALL authors)

Please check all that apply

__✔__ New author(s) have been added (in addition to this form, all new authors must complete the copyright transfer agreement and conflict of interest disclosure.

_____ Change in order of authorship.

_____ An author wishes to remove his/her name. An author's name may only be removed his/her own request and a letter signed by the author should accompany this form

Manuscript Number STROKE/202(

Manuscript Title Metabolic traits and stroke risk in individuals of African ancestry: Mendelian ranc

Former Authorship

Please list ALL AUTHORS in the same order as the original submission. For more than 12, use an extra sheet.

Print Name		Print Name	
Name (1)	Segun Fatumo	Name (7)	Mariam Nakabuye
Name (2)	Ville Karhunen	Name (8)	Opeyemi Soremekun
Name (3)	Tinashe Chikowore	Name (9)	Iyas Daghlas
Name (4)	Toure Sounkou	Name (10)	David K. Ryan
Name (5)	Brenda Udosen	Name (11)	Amybel Taylor
Name (6)	Chisom Ezenwa	Name (12)	

New Authorship

All authors must sign below agreeing to the changes in authorship. The authorship order must reflect the authorship order of the manuscript.

Name		Signature	Date
Name (1)	Segun Fatumo		
Name (2)	Ville Karhunen		
Name (3)	Tinashe Chikowore		
Name (4)	Toure Sounkou		
Name (5)	Brenda Udosen		
Name (6)	Chisom Ezenwa		
Name (7)	Mariam Nakabuye		
Name (8)	Opeyemi Soremekun		
Name (9)	Iyas Daghlas	[signature]	2/11/21
Name (10)	David K. Ryan		
Name (11)	Amybel Taylor		
Name (12)	Amy M. Mason		

Please scan and email to stroke@strokeahajournal.org.
