# Supplementary material for: Metabolic Traits and Stroke Risk in Individuals of African Ancestry: Mendelian Randomization Analysis
Source: Stroke. 2021 Jun 3;52(8):2680–4. doi: 10.1161/STROKEAHA.121.034747 (PMC8312569; doi:10.1161/STROKEAHA.121.034747)
Supplement: Supplementary file 14 [file str-52-2680-s014.pdf]

Change of Authorship Form

(Must be completed and signed by ALL authors)

Please check all that apply

✔ New author(s) have been added (in addition to this form, all new authors must complete the copyright transfer agreement and conflict of interest disclosure.

_____ Change in order of authorship.

_____ An author wishes to remove his/her name. An author's name may only be removed his/her own request and a letter signed by the author should accompany this form

Manuscript Number STROKE/2020/034093

Manuscript Title Metabolic traits and stroke risk in individuals of African ancestry: Mendelian randomization analysis

Former Authorship

Please list ALL AUTHORS in the same order as the original submission. For more than 12, use an extra sheet.

**Print Name**	**Print Name**
Name (1) Scott M. Damrauer	Name (7) Dipender Gill
Name (2) Marijana Vujkovic	Name (8)
Name (3) Keith L. Keene	Name (9)
Name (4) Myriam Fornage	Name (10)
Name (5) Marjo-Riitta Järvelin	Name (11)
Name (6) Stephen Burgess	Name (12)

New Authorship

All authors must sign below agreeing to the changes in authorship. The authorship order must reflect the authorship order of the manuscript.

Name	Signature	Date
Name (1) Scott M. Damrauer	Signature	Date
Name (2) Marijana Vujkovic	Signature	Date
Name (3) Keith L. Keene	Signature	Date
Name (4) Myriam Fornage	Signature	Date 2/10/2021
Name (5) Marjo-Riitta Järvelin	Signature	Date
Name (6) Stephen Burgess	Signature	Date
Name (7) Dipender Gill	Signature	Date
Name (8)	Signature	Date
Name (9)	Signature	Date
Name (10)	Signature	Date
Name (11)	Signature	Date
Name (12)	Signature	Date

Please scan and email to stroke@strokeahajournal.org.
